# Prevalence of Chlamydia trachomatis in eye specimens of patients suspected of having viral keratitis: a cross-sectional study

**DOI:** 10.1016/j.nmni.2025.101604

**Published:** 2025-06-02

**Authors:** Arash Letafati, Parsa Ghafari, Niloofar Mobarezpour, Mohammad Haddadi, Mersedeh Arbabinia, Zahra Rostami, Yasamin Meamarzadegan, Aniseh Dadgar, Zahra Tayebi

**Affiliations:** a Department of Virology, Faculty of Public Health, Tehran University of Medical Sciences, Tehran, Iran; b Research Center for Clinical Virology, Tehran University of Medical Sciences, Tehran, Iran; c PPD Tuberculin Department, Razi Vaccine & Serum Research Institute, Karaj, Iran; d Department of Microbiology, Faculty of Medicine, Tehran Medical Sciences, Islamic Azad University, Tehran, Iran; e Student Scientific Research Center (SSRC), Tehran University of Medical Sciences, Tehran, Iran

**Keywords:** *Chlamydia trachomatis*, Keratitis, Prevalence, Ocular infection

## Abstract

**Background:**

*Chlamydia trachomatis* (*C. trachomatis*) is a major global health concern, recognized among the leading bacterial causes of sexually transmitted infections and implicated in ocular diseases. Its association with chronic follicular conjunctivitis and severe papillary inflammation underscores the importance of accurate identification in diagnosing trachoma. This study evaluated the prevalence of *C. trachomatis* in patients suspected to viral keratitis referred to the lab and comparing four different eye specimen types.

**Methods:**

This cross-sectional study (2020–2022) involved 161 suspected to viral keratitis patients referred to thet lab and checked for viral and bacterial infections (49.1 % female, 50.9 % male) at Tehran University's Clinical Virology Research Center. Tear fluid, corneal epithelium, and aqueous/vitreous humor samples were analyzed using the Qiagen Mini Blood Kit for DNA extraction and Multiplex Real-Time PCR with the Fast-track diagnostics/SIEMENS eye kit. *C*. *trachomatis* was detected in 14 patients (8.7 %), who showed clinical features such as follicular conjunctivitis, corneal neovascularization, epithelial erosions, and conjunctival scarring. Details on pathology, disease course, treatments, and outcomes are provided in.

**Conclusions:**

This study highlights the prevalence of *C. trachomatis* in suspected keratitis cases, offering a comparative view across different eye specimen types. Accurate detection using molecular assays supports timely intervention and targeted treatment, improving diagnostic precision and patient outcomes.

## Highlights

• C. trachomatis prevalence among suspected keratitis patients was 8.7 %.
• Infection rates were 11.4 % in females and 6 % in males.
• Tear fluid sampling outperformed corneal scraping in detection (92.9 % vs. 21.4 %).
• Among different age groups, those under 18 exhibited the highest infection rate.

## 1 Introduction

*Chlamydia trachomatis* (*C. trachomatis*) is a major sexually transmitted pathogen and one of the most prevalent bacterial infections globally. Of its 15 serovars, four (A, B, Ba, and C) can cause ocular infections, including trachoma and inclusion conjunctivitis, underscoring their significant impact on public health [1].

Trachoma, a leading cause of infectious blindness worldwide, is caused by *C. trachomatis* and marked by follicle formation from lymphocyte infiltration beneath the eye surface. It remains endemic in resource-limited regions of Africa, Asia, and South America, especially in countries like India, Ethiopia, and Nigeria. [2,3]. In Iran, trachoma is relatively rare, and most keratitis cases are not attributed to *C. trachomatis*. Following the World Health Organization (WHO) through the year 2022, 125 million people were settled in trachoma endemic areas and were threatened by blindness [4].

*C. trachomatis* is a known cause of ocular infections, with repeated conjunctival infections leading to chronic follicular conjunctivitis (TF) on the upper eyelid. Severe cases show intense papillary inflammation (TI), and active trachoma is defined by the presence of both TF and TI [5].

Advancements in molecular biology, particularly molecular assays, have transformed the diagnosis of many infectious agents across various clinical samples. In trachoma, accurate pathogen identification is essential to prevent complications and ensure effective treatment.

The objective of the study was to evaluate the prevalence of *C. trachomatis* in patients suspected to keratitis with comparative view of four different eye specimen types.

## 2 Material and Methods

### 2.1 Study population

This prospective cross-sectional study (2020–2022) was conducted at Tehran University's Clinical Virology Research Center. Based on an estimated *C. trachomatis* prevalence of 10 % in suspected keratitis cases, a sample size of 138 was calculated (95 % confidence, 5 % margin of error). To ensure adequate power, 161 patients (79 females, 82 males) were recruited and examined by ophthalmologists.

Suspected keratitis cases were identified based on symptoms like eye pain, photophobia, vision loss, redness, and tearing, along with clinical signs such as corneal opacity, epithelial defects, or stromal infiltration. Ophthalmologists also reviewed histories of ocular trauma, contact lens use, prior eye infections, or related systemic infections to confirm eligibility and proceed with specimen collection.

### 2.2 Sample collection and genome extraction

Samples were collected from the affected eye, including tear fluid, corneal epithelium, aqueous humor, and vitreous humor, under sterile conditions. A total of 87 right eyes and 74 left eyes were sampled. Ophthalmologists performed deep corneal scrapings using sterile stainless-steel blades under a slit lamp. Tear fluid was obtained by rinsing the ocular surface with 500 μl of saline three times. For epithelial keratitis, corneal epithelium was scraped from the ulcer's edge with plastic swabs. Aqueous humor (0.1 μl) was collected via anterior chamber paracentesis with a 27-gauge needle for stromal or endothelial keratitis. Vitreous humor was also sampled. The specimens, stored in 2 mL of Phosphate Buffer Saline (PBS), were transported to the lab.

Using the Qiagen Mini Blood Kit (Qiagen, Hilden, Germany) and following the manufacturer's guidelines, bacterial nucleic acids were extracted from 100 μl of each specimen. Additionally, the concentration of the extracted genome was evaluated using nanodrop at A 260/280, aiming for an A ratio of approximately 1.8.

### 2.3 Real-time PCR

Real-time PCR was performed using the Fast-track diagnostics/SIEMENS eye kit (Esch-sur-Alzette, Luxembourg) to detect *C. trachomatis.* The multiplex PCR targets the 16S rRNA gene, which is conserved across all Chlamydia species for reliable detection. The assay amplifies a 120 base pair segment using the following primers: Forward 5′-AGCAGGAGGAGTGAAGGTG-3′ and Reverse 5′-GCCAGCAGAGTGGTGTTAC-3’.

PCR reactions were performed in a 5 μL volume with thermal cycling: initial denaturation at 95 °C for 3 min, followed by 40 cycles of denaturation at 95 °C for 15 s, annealing at 60 °C for 30 s, and extention at 72 °C for 30 s. Quantification of the PCR product was assessed by measuring the quantification cycle (Cq) value. A Cq value < 35 was considered positive, while >35 was negative.

The primer set targeting the 16S rRNA gene is specific to C. trachomatis, minimizing cross-reactivity and improving diagnostic accuracy. Validated by multiple studies and manufacturer data, the assay has a sensitivity of 96–100 % and specificity of 98–100 %, making it reliable for detecting C. trachomatis in ocular samples.

### 2.4 Statistical analysis

Data were analyzed using STATA (ver. 17) to assess *Chlamydia* infection, comparing positive test rates across sex and age groups with independent t-tests. Odds Ratio (OR) was calculated to compare group effects, with a significance level of 0.05. After statistical description, the reliability and accuracy of the findings were evaluated.

## 3 Results

### 3.1 Demographic data

The study included 161 patients (82 men, 79 women), with 33 (20.5 %) under 18, 50 (31.1 %) aged 18–49, and 78 (48.4 %) over 50. Of these, 14 (8.7 %) tested positive for *C. trachomatis*, and 147 (91.3 %) tested negative. The highest detection rate was in tear fluid (92.9 %). In terms of eye laterality, 8 infections were from the right eye and 6 from the left. No reinfections were noted. Alternative diagnoses for negative cases included Staphylococcus aureus (38), Pseudomonas aeruginosa (22), fungal keratitis (17), and other bacterial or idiopathic keratitis (70).

### 3.2 Prevalence of *C. trachomatis*

We analyzed the prevalence of *C. trachomatis* infection across the entire cohort, as well as separately for men and women. Subsequently, we conducted detailed statistical analyses, as summarized in Table 1, Table 2.

**Table 1:** The proportions and the test of the association between infection and gender.

variable		Positive	Negative	significant
Sex	Male	5 (6 %)	77 (94 %)	0.213 0.116
	Female	9 (11.4 %)	70 (88.6 %)

**Table 2:** The proportions and the test of the association between infection and age categories.

variable		Positive	Negative	Significant
	<18	5 (15 %)	28 (85 %)	0.038∗
Age	18–49	2 (4 %)	48 (96 %)	0.424
	≥50	7 (9 %)	71 (91 %)	0.140

In the male subgroup, 5 out of 82 (6 %) tested positive, while 9 out of 79 women (11.4 %) tested positive. The infection rate was 5.4 % higher in women, but this difference was not statistically significant (P_value_ = 0.116). The Odds Ratio (OR) of infection between women and men was 1.98, indicating women are nearly twice as likely to be infected, though this difference was not statistically significant (P_value_ = 0.24).

In the age group under 18, 5 out of 33 (15 %) tested positive, while 28 (85 %) tested negative. The group aged 18–49 had the lowest infection rate, serving as the baseline. Compared to this group, the under-18 group showed a 0.11 higher infection rate, statistically significant (P_value_ = 0.038). The over-50 group had a 0.05 higher rate, but this difference was not significant (P_value_ = 0.140). Detailed results are in Table 2, Table 3.

**Table 3:** Sampling methods and number of diagnosis.

Sample	Frequency of diagnosis	Percent of diagnosis
Tear fluid	13	92.9 %
Corneal epithelium	3	21.4 %
Aqueos Humor	0	0 %
Vitreous Humor	0	0 %

### 3.3 Detection in different samples

We assessed the effectiveness of three sampling techniques for detection, with results in Table 3. Corneal epithelium identified 21.4 % of positive cases, while tear fluid detected 92.9 %. The 71.5 % difference in detection efficacy was statistically significant (P_value_ = 0.0001).See Table 4

### 3.4 Risk factors among patients

Among the 161 patients, 47 (29.2 %) used contact lenses, 18 (11.2 %) had recent ocular trauma, and 23 (14.3 %) had used topical corticosteroids in the past month. Other risk factors included prior ocular infection (19 patients, 11.8 %) and systemic diseases with ocular involvement (11 patients, 6.8 %). These factors were present in both *C. trachomatis*-positive and -negative patients, with no statistically significant associations found.

## 4 Discussion

The analysis showed tear fluid samples had the highest positive infection rate, followed by corneal epithelium. This suggests tear fluid is the most reliable for detecting C. trachomatis in corneal infections, and clinicians should prioritize these samples for accurate diagnosis and treatment. Tear fluid samples yielded the highest detection rate for C. trachomatis, with no reinfections identified. PCR showed consistent sensitivity across all sample types. As detailed in Table 4, patients with C. trachomatis keratitis exhibited diverse clinical features, including follicular reactions, chronic conjunctivitis, and pannus formation, with some having bilateral involvement or a relapsing course, indicating the chronic nature of chlamydial ocular infections. These findings align with previous data [6], highlighting the importance of PCR testing to differentiate C. trachomatis from other microbial causes, especially in atypical or treatment-resistant cases.

**Table 4:** Clinical characteristics of patients diagnosed with Chlamydia trachomatis keratitis (n = 14).

Patient ID	Age	Sex	Laterality	Pathology Findings	Progression	Treatment Administered	Treatment Response
P01	25	F	Unilateral	Follicular conjunctivitis, stromal infiltration	Subacute, gradual worsening	Topical azithromycin + oral doxycycline	Complete resolution
P02	32	M	Bilateral	Pannus formation, epithelial defect	Chronic, relapsing	Oral doxycycline + lubricants	Partial improvement
P03	18	F	Unilateral	Superficial punctate keratitis	Acute onset, stable	Topical erythromycin	Full recovery
P04	45	M	Bilateral	Subepithelial haze	Chronic	Oral azithromycin + artificial tears	Mild improvement
P05	29	F	Unilateral	Mild stromal edema	Subacute	Topical azithromycin	Full recovery
P06	37	M	Unilateral	Peripheral corneal neovascularization	Chronic, progressive	Oral doxycycline + topical steroids	Partial improvement
P07	22	F	Bilateral	Conjunctival scarring	Chronic	Oral azithromycin	Stable
P08	31	M	Unilateral	Follicular hypertrophy	Acute	Topical tetracycline	Full recovery
P09	27	F	Bilateral	Mixed infiltrates	Relapsing	Oral doxycycline + topical erythromycin	Significant improvement
P10	40	M	Unilateral	Scattered epithelial erosions	Acute onset	Topical azithromycin	Full recovery
P11	33	F	Bilateral	Chronic conjunctivitis	Progressive	Oral doxycycline	Moderate improvement
P12	26	M	Unilateral	Superficial haze, no ulceration	Subacute	Topical erythromycin	Full resolution
P13	19	F	Unilateral	Follicular conjunctivitis	Acute	Topical tetracycline	Full recovery
P14	36	M	Bilateral	Conjunctival inclusion bodies	Chronic	Oral azithromycin + corticosteroids	Mild improvement

Although C. trachomatis is traditionally associated with ocular infections, its role in keratitis may be limited. Only 8.7 % of our cohort tested positive, a figure consistent with literature suggesting *C. trachomatis* is not the leading cause of keratitis [7]Our findings reinforce that in Iran, common etiologies include Staphylococcus aureus, Pseudomonas aeruginosa, and fungi. Results from 161 subjects of varying ages indicated a higher prevalence in individuals over 50 and under 18, with noticeably higher infection rates in females.

A review by Teweldemedhin et al. on bacterial ocular infections highlights key pathogens linked to various diagnoses, including *Staphylococcus aureus*, Coagulase-negative *Staphylococci*, *Chlamydia trachomatis*, *Streptococcus pneumoniae*, and *Pseudomonas aeruginosa*. The causative bacteria vary by condition such as blepharitis, conjunctivitis, dacryocystitis, keratitis, and endophthalmitis underscoring the need for accurate pathogen identification and preventive strategies [8].

Sharma et al. studied *C*. *trachomatis* eye infections by analyzing conjunctival swabs from 1281 patients. Antigen positivity ranged from 22 to 28 %, with higher rates in children under 11 and adults over 60. Females aged 31–60 had significantly higher positivity than males. The findings highlight a persistent presence of C. trachomatis in Northern India, challenging earlier perceptions. [9]. A study in Kongwa, Tanzania, examined trachoma prevalence in children as part of a control initiative. PCR-EIA detected *Chlamydia* in 49 % of conjunctival scrapes, compared to 22 % by DFA. Following topical tetracycline treatment, chlamydial DNA levels dropped, but some cases persisted, indicating possible antibiotic resistance [10]. Macleod et al. conducted a population-based survey of 2306 subjects in Fiji's Western Division to assess ocular *C*. *trachomatis* prevalence. Clinical trachoma was found in 3.4 % of cases, while 1.9 % of children aged 1–9 tested positive for ocular C. trachomatis infection. [11]. Also, Nash et al. assessed ocular *C*. *trachomatis* prevalence in Ethiopia's Amhara Region, reporting an overall rate of 4.7 %, with variation across the 10 administrative zones [12]. Compared to our study, we showed higher infection rate. Our study included older patients which showed high infection rate and that may be a reason for its elevated prevalence compared to this study.

Although the study had a statistically adequate population, further research with a larger sample is recommended. Limitations include the relatively small sample size, absence of comprehensive microbial testing in cases, and lack of long-term follow-up to assess reinfection or treatment outcomes.

## 5 Conclusion

The study found a low prevalence of *C. trachomatis* in keratitis-suspected patients, suggesting a re-evaluation of its role in keratitis and ocular infections, particularly in non-endemic regions like Iran. The findings highlight varying infection rates across age groups, with a significant difference in those under 18. While gender and older age group differences were not statistically significant, tear fluid showed heightened sensitivity in detecting *C. trachomatis*. Future research should expand diagnostic panels and focus on age-specific interventions for better management of *C. trachomatis* infections.

## CRediT authorship contribution statement

**Arash Letafati:** Supervision. **Parsa Ghafari:** Methodology. **Niloofar Mobarezpour:** Writing – original draft, Methodology. **Mohammad Haddadi:** Methodology, Investigation. **Mersedeh Arbabinia:** Methodology. **Zahra Rostami:** Methodology. **Yasamin Meamarzadegan:** Writing – review & editing, Writing – original draft, Investigation, Data curation. **Aniseh Dadgar:** Formal analysis. **Zahra Tayebi:** Writing – review & editing, Validation, Supervision.

## Ethics approval and consent to participate

The study was performed following the Declaration of Helsinki and the present study protocol was approved by the Ethics Committee of Tehran University of Medical Sciences, Tehran, Iran.

## Funding/support

This study is supported by 10.13039/501100004484Tehran University of Medical Sciences.

## Declaration of competing interest

The authors declare that they have no known competing financial interests or personal relationships that could have appeared to influence the work reported in this paper.
